# Conference Report: WORKSHOP ON POLYMER BLENDS Gaithersburg, MD April 20–21, 1992

**DOI:** 10.6028/jres.097.021

**Published:** 1992

**Authors:** Edmund A. Di Marzio, Charles C. Han

**Affiliations:** National Institute of Standards and Technology, Gaithersburg, MD 20899

## 1. Introduction

The authors are aware that virtually every government laboratory and agency is grappling with the problem of technology transfer to American industry. This activity is in response to the Federal Technology Transfer Acts (FTTA) of 1986 and 1989, which were conceived in an attempt to make American industry more competitive world wide. These acts in turn are a response to the fact that so many American industries have not maintained their own research efforts even in the very areas that are needed for their own company’s survival. This simultaneous blossoming of effort in the various federal laboratories will bear no fruit unless practicing scientists become involved. The workshop on polymer blends was conceived to examine whether an effort involving technology transfer and collaboration in the area of polymer blends is feasible.

Logically there are two necessary conditions. First, industry must identify common needs in the area of polymer blends. Second, the required expertise must exist at NIST to address these needs.

Concerning the second necessary condition we remark that during the first part of the workshop, the Polymer Blends and Solutions group with some help from others in the Polymers Division and from the Reactor Division, presented 3 invited talks and 11 posters (see Appendix B). These presentations along with laboratory site visits to sbc different NIST facilities and 30 reprints and preprints of work on polymer blends done at NIST over the past year provide ample evidence of NIST proficiency in polymer blends.

The companies (all of which conduct research on polymer blends) each admitted that their approach to problems was practical and not focussed on the underlying science base. Industry attendees agreed that knowledge of the basics would be useful, but that they seldom had the time and resources to address basic questions. There definitely is a need for the underlying science base which demonstrates that the two conditions for a fruitful collaboration between industry and NIST are met.

The above remarks suggest a strategy. Have a model system that companies and NIST both agree would be of interest and have NIST study it in depth, with both experiments and theory. Possible choices are given in the “Proposed NIST/Industry Consortium on Polymer Blends” in Appendix A.

## 2. Summary of the Scientific Part of the Workshop

The eight abstracts of the invited talks and the 17 abstracts of the poster presentations are given in Appendix B and provide a summary of the scientific portion of the meeting.

A consensus emerged from these presentations and discussions that the most important scientific questions concern the nature of the interface. Specifically, the growth of the interface with time during spinodal decomposition and ripening, the influence of interfacial modifiers on blend properties, the design of compatibilizing agents, and the improvement of toughening agents by modifying their interface are all very difficult problems of very great interest to industry.

## 3. Scope of the Problem

This listing serves to demonstrate the degree of complexity of polymer blends technology and how much remains to be done.

1. Physical State; Amorphous-Amorphous, Amorphous-Crystal, and Amorphous-Liquid crystal Blends; Crystal-Crystal and Liquid Crystal-Liquid Crystal Blends
2. Phase Separation in Blends
  - Flow induced mixing-demixing
  - Separation during polymerization, cross-linking, or gel formation.
  - Ripening-late stage growth
3. Compatibilizers
  - Copolymers and block copolymers
  - Hydrogen bonding between A and B molecules, ions
  - Chemically modify one or both polymers, grafting, transesterification, reactive processing
  - Addition of third component
4. Mechanical Properties
  - Modulus, mechanical strength
  - Failure, fracture, crazing
  - Viscoelastic properties, processibility
  - Physical ageing
5. Morphological Studies, Structure/Property Relations
6. Thin Film Blends
7. Experimental Methods
  - Light scattering (LS), temperature jump and shear LS
  - Small Angle Neutron Scattering (SANS), SANS with Shear
  - FTIR
  - Infrared dichroism
  - Microscopy
  - Small Angle X-ray Scattering (SAXS)
8. Blends on Macro, Micro, and Nanometer Length Scales
9. Miscellaneous
  - Ceramic-polymer blends
  - Metal-polymer blends
  - Stabilizers in blends
  - Wetting transition
  - Photodegradation
  - Blend processing monitoring
  - Blends as resins in polymer composites
  - Blends as route to gels, aero-gels, and low density materials

## 4. Industry’s Perspective

Those industries with actual profitable polymer blends product lines were concerned mainly with maintaining quality and perhaps making incremental improvements. Those who were seeking to expand their product lines were more interested in novel materials.

Rubber toughened blends were of interest to several groups and interfacial compatibilizers that would bind the rubber to the rest of the blend matrix were of intense interest. As mentioned above there was a general consensus that polymer interface problems were of paramount interest.

All those companies represented at the work-shop, except for one (represented by a postdoctoral associate), responded positively. Company representatives will provide all necessary documents and the consortium proposal for evaluation by their management. As a general statement we can say that the industrial scientists favored the formatiom of a NIST/industry consortium.

The managers were quite willing to give funds for specific items, but were wary of allotting money to a consortium without specific research or other activities identified in advance. We think this may be due to past experiences with industry-university consortia where company contributions were general funds over which they had little control. This obviously means that the model systems proposed for study in the consortium must be agreed to before the actual funding takes place. Perhaps we should not use the word consortium fee.

## 5. NIST/Industry Collaboration

The workshop succeeded in fostering direct interactions between industry and NIST in the general area of polymer blends. Strong support for the polymer blends work at NIST was given by the industrial representatives. The industry representatives agreed to comment on the proposed consortium (Appendix A) after discussions with their management. Comments were made by several industrial representatives that a NIST programmatic initiative on polymer blends/alloys should be seriously considered due to the needs and size of industry.

## 6. Summary

This report describes a 2 day workshop on polymer blends held at the National Institute of Standards and Technology on April 20–21, 1992. There were 49 attendees, 22 from industry, 4 from universities and 23 from NIST. The objectives were threefold. First, to illustrate the importance and vitality of the subject 8 invited lectures and 17 posters were presented, and were the subject of lively discussions among attendees. Second, to show that NIST is highly competent in polymer blends research three NIST scientists gave invited talks and 11 of the 17 poster presentations were given by NIST employees. Also, preprints and reprints of 30 NIST publications were available to the industry representatives. Third, to ascertain what Industry perceives as needs in the area of polymer blends a 2 hour session titled Future Directions, Collaborative Efforts and Consortium was conducted. This session was co-chaired by Peter Juliano of GE and by Charles Han of NIST. Panel members were Michael Blaney of NIST, David Lohse of Exxon, Donald Paul of University of Texas and Arthur Yang of Armstrong World industries. The ultimate goal of the workshop is the formation of a NIST/industry consortium. A proposal for a consortium was presented and is reproduced in Appendix A. Laboratory visits to six separate facilities at NIST concluded the work-shop.

## 7. Appendix A. Proposed NIST/Industry Consortium on Polymer Blends/Alloys

### POLYMER BLENDS AND SOLUTIONS GROUP POLYMERS DIVISION NATIONAL INSTITUTE OF STANDARDS AND TECHNOLOGY (Revised May 18,1992)

This is a proposal to form a NIST/industry consortium on polymer blends/alloys. The purpose is to stimulate frontier research in polymer blends/alloys processing with special emphasis on interfacial structure characterization and formation. Also, the purpose is to establish a dialogue and share information between NIST and U.S. industrial companies on new developments in measurement techniques, in analysis procedures, and in the science and technology relating to the processing of polymer blends/alloys.

The proposed plan is subject to further discussion, modification and approval of the consortium. The NIST/industry consortium on polymer blend alloys will consist of NIST and at least three U.S. companies.

- Product and Benefit
  Since the toughening and reinforcement through (micro-)phase separated structure is the main emphasis in polymer blends/alloys processing, the interfacial structure characterization and formation are priority R/D interests, and the emphasis of the research plan for this consortium. Specifically:
  1. To study in-depth and publish results on a blend system, with emphasis on the effect of interfacial modification. This includes the domain and interfacial structure formation, characterization, compatiblization, mixing/demixing kinetics under the influence of the interface modification.
  2. To share knowledge of current science/technology on polymer blends, measurement/characterization techniques, and analysis procedures.
  3. Access to specialty instruments available at NIST, and to gain knowledge of the operation, function and also limitation of these instruments.
  4. To learn of technology and techniques available from NIST staff that might impact on further development of company specific R/D projects.
  5. Coexclusive rights to patents arising from the consortium’s research project.
- Proposed Plan
  The initial project will be an in depth study on a nonproprietary blend system. Through this study company scientists become familiar with science and technology, measurement techniques, and analysis procedures available at NIST. Generic technology about interfacial structure formation and characterization can be developed which may be applied to company specific blends/alloys development.
  1. Possible Blend Systems for Initial Study:
    - Deuterated polystyrene/polybutadiene with styrene-butadiene block copolymer as interfacial modifier.
    - Deuterated polybutadiene/polyisoprene with butadiene-isoprene copolymer as interfacial modifier.
    - Ethylene-propylene copolymer/polypropylene (or polyethylene) blend with corresponding block (grafted) copolymer as interfacial modifier.
    The final decision on the selection from these three systems for initial study will be decided by the majority of member companies of the consortium.
  2. Properties to be Studied:
    - Preliminary study of the equilibrium and kinetic phase behavior of the selected system with and without the block copolymer interfacial modifier.
      - Equilibrium phase diagram, including temperature, *T*, and composition, *ϕ*, dependence of the χ-parameter (obtained from free energy function of mixing). Construct coexistence curves and spinodal curves for various molecular weights of the blend system.
      - Kinetics *of* phase separation (spinodal decomposition) in the early and late stages.
    - Shear mixing effect under the influence of interfacial modifier.
      Structure growth and/or stablization and compatiblization with the interfacial modifier after cessation of flow.
    - Interfacial profile characterization through small angle neutron scattering (in the Porod law region at wider angles) and through neutron reflectivity experiments.
  3. Techniques To Be Used:
    Small angle neutron scattering (SANS), with/without (w/o) shear dependence and w/o time resolved measurements.
    Light Scattering (LS), w/o temperature jump (or quench), w/o shear dependence, w/o time resolved, and simultaneous shear and quench experiments.
    Neutron reflectivity for interfacial profile analysis under various equilibrium and quenched conditions.
  4. Other Blend Systems:
    Each company will be given 2 days/yr of additional SANS time upon request to try out company specific, but nonproprietary systems. If reflectometer time is available this may be substituted for SANS time.
- Contributions from Participants
  1. From NIST
    		Estimated Cost
    (A)	One person/yr	$150 K
    (B)	20–25 days of SANS instniraent/yr	$ 75 K
    (C)	20–30 days of LS instniment/yr	$ 30 K
    	Total	$255 K
  2. From Companies
    - Active participation is the key to the consortium. Estimated 4 weeks of technical staff time/company/yr
    - Experimental fee of $15 K/corapany/yr
    - Donation of materials or sharing cost for special synthesis, if necessary.
    - Company’s option of sending Research Associate to work at NIST.
- Duration and Target Starting Date of the Consortium
  1. Duration: 3 years with annual evaluation and review.
  2. Target starting date: October 1, 1992.
- R/D on Company Specific Project:
  During or after this 3 year project, a member company may initiate separate projects specific to the company’s R/D interests. This will be possible under the following two mechanisms.
  1. Proprietary use of NIST facility/instrument.
  2. Through a Separate Cooperative Research and Development Agreement and (if necessary) Nondisclosure Agreement.

## 8. Appendix B. Invited and Contributed Talks

### 8.1 Invited Presentations

1. Strategies for Compatiblization of Polymer Blends
  D. R. Paul
  The University of Texas at Austin,
  Austin, Texas
2. Neutron Scattering from Polymers
  Boualem Hammouda
  National Institute of Standards and Technology,
  Gaithersburg, MD
3. Miscibility of Saturated Hydrocarbon Polymer Blends
  N. P. Balsara
  Polytechnic University, Brooklyn, NY
  L. J. Fetters
  University of Athens,
  Athens, Greece
  D. J. Lohse
  Exxon Research and Engineering Co.,
  Linden, NJ
  C. C. Han
  National Institute of Standards and Technology,
  Gaithersburg, MD
  W. W. Grassley, R. Krishnamoorti
  Princeton University,
  Princeton, NJ
4. Rheological and Mechanical Properties of Antiplasticized and Rubber Toughened Polycarbonates
  Robert P. Kambour
  General Electric,
  Schenectady, NY
5. Fundamentals of Extrusion Compounding of Polymer Blends
  Chi-Kai Shih
  DuPont,
  Wilmington, DE
6. Ordering in Block Copolymers, and Polymer Blends
  Under Shear
  M. Muthukumar
  University of Massachusetts,
  Amherst, MA
7. Phase Behavior of Polymer Blends Under Steady Shear
  Flow and After Cessation of Flow
  Charles C. Han
  National Institute of Standards and Technology,
  Gaithersburg, MD
8. Measurement of NM-Range Domain Dimensions and Estimation of Stoichiometries in Phase-Separated Polymer Blends Using Solid-State Proton NMR
  D. L. VanderHart
  National Institute of Standards and Technology,
  Gaithersburg, MD

### 8.2 Poster Presentations

1. Shear-SANS and -Light Scattering Instruments for In-Situ Mixing/Demixing Studies of Polymer Blends and Solutions
  Y.-B. Ban, A. I. Nakatani, and C. C. Han
  National Institute of Standards and Technology,
  Gaithersburg, MD
2. Facilities for Small-Angle X-Ray Scattering Measurements
  John D. Barnes
  National Institute of Standards and Technology,
  Gaithersburg, MD
3. Small Angle Neutron Scattering Studies of Single Phase IPNS
  Barry J. Bauer and Robert M. Briber
  National Institute of Standards and Technology,
  Gaithersburg, MD
  Shawn Malone and Claude Cohen
  Cornell University,
  Ithaca, NY
4. The Morphology of Poly(Vinylidene Fluoride)
  Ciystallized from Blends of Poly(VinyIidene Fluoride)
  and Poly(Ethyl Acrylate)
  Robert M. Briber and Freddy A. Khoury
  National Institute of Standards and Technology, Gaithersburg, MD
5. Image Analysis of the Late Stages of Phase Separation in Polymer Blends
  Robert M. Briber
  University of Maryland,
  College Park, MD
  Eugene Gholz and Shiming Wu
  National Institute of Standards and Technology,
  Gaithersburg, MD
6. Phase Behavior of Poly Amino Acids: Solutions and Blends
  M. D. Dadmun and C. C. Han
  National Institute of Standards and Technology,
  Gaithersburg, MD
  M. Muthukumar
  University of Massachusetts,
  Amherst, MA
7. The Surface Tension of Polymer Blends
  Gregory T. Dee and Bryan B. Sauer
  DuPont,
  Wilmington, DE
8. Critical Point Viscosity of Polymer Blends
  Jack Douglas
  National Institute of Standards and Technology,
  Gaithersburg, MD
9. A New Twist in Blending Polymer Compounds
  Semih Erhan
  Albert Einstein Medical Center,
  New York, NY
10. Static and Dynamic Critical Behavior of a Low Molecular Weight Binary Polymer Blend
  D. W. Hair, E. K. Hobbie, J. Douglas, and C. C. Han
  National Institute of Standards and Technology,
  Gaithersburg, MD
11. Morphology Effects in the Fracture Behavior of Two Phase Thermoset Polymers
  Donald Hunston
  National Institute of Standards and Technology,
  Gaithersburg, MD
12. Small Angle Neutron Scattering (SANS) Instruments at NIST’s Cold Neutron Research Facility
  C. Glinka, B. Hammouda, J. Barker and S. Kruger
  National Institute of Standards and Technology,
  Gaithersburg, MD
  C. C. Han
  National Institute of Standards and Technology,
  Gaithersburg, MD
13. Inversion of Phase Diagram in Deuterated Polybutadiene and Protonated Polybutadiene Blend
  Hiroshi Jinnai, Hirokazu Hasegawa and Takeji Hashimoto
  Kyoto University,
  Kyoto 606, Japan
  Charles C. Han
  National Institute of Standards and Technology,
  Gaithersburg, MD
14. Crystallization Effect on Morphology of Poly(Aiyl Ether Ketones) and Poly(Ether Imide) Miscible Blends
  Benjamin S. Hsiao, Bryan B. Sauer and John Van Alsten
  DuPont,
  Wilmington, DE
15. Shear Rate Dependence of Phase Separation Kinetics After Cessation of Shear
  A. I. Nakatani, Y. B. Ban, and C. C. Han
  National Institute of Standards and Technology
  Gaithersburg, MD
16. Neutron Reflectivity from Polymer Surfaces and Interfaces
  Sushil K. Satija, J. F. Ankner, and C. F. Majkrzak
  National Institute of Standards and Technology
  Gaithersburg, MD
17. Competition of Phase Separation and Transesterflcation in d-PoIycarbonate/co-Polyester Blends
  Hichang Yoon and C. C. Han
  National Institute of Standards and Technology,
  Gaithersburg, MD
  Yi Feng
  University of Connecticut,
  Storrs, CT

